# Optimizing electrocoagulation for poultry slaughterhouse wastewater treatment: a fuzzy axiomatic design approach

**DOI:** 10.1007/s11356-024-33069-4

**Published:** 2024-04-16

**Authors:** Nazire Pınar Tanatti, Mesut Sezer

**Affiliations:** 1 Department of Environmental Protection Technologies, Sakarya University of Applied Sciences, 54100 Sakarya, Turkey; 2 Department of Environmental Engineering, Kocaeli University, 41001 Kocaeli, Turkey

**Keywords:** Fuzzy axiomatic design, Electrocoagulation, Blood, Poultry slaughterhouse wastewater, Aluminum electrode, Iron electrode

## Abstract

White meat consumption is increasing day by day, and accordingly, there is an increase in the amount of wastewater resulting from the processes. Today, the reuse of wastewater has become a goal within the scope of the Green Deal. For this reason, wastewater treatment with high pollution and volume has gained importance. In this study, the fuzzy axiomatic design (FAD) method, one of the multi-criteria decision-making methods, has been used. With this method, coagulation, electrocoagulation (EC), dissolved air flotation (DAF), and anaerobic treatment alternatives preferred in poultry slaughterhouse wastewater (PSW) treatment were compared with each other and their information contents were calculated. The information content from the smallest to the largest is EC, DAF, coagulation, and anaerobic treatment, respectively. This treatment method was chosen because the smallest information content is in electrocoagulation. EC was applied to bloody PSW containing 1% blood by volume. The effectiveness of Fe and Al electrodes for PSW treatment in the batch EC reactor has been compared. The effective surface areas of 2 anodes and 2 cathodes connected bipolarly in the processes are 288 cm^2^. The electrolyte, pH, time, and current density effects on energy consumption were also investigated. The optimum conditions for Al and Fe electrodes were found to be 0.5 g·L^−1^ NaCl concentration, pH 5, 0.639 mA·cm^−2^ current density, and 5 min time. Under optimum conditions for the Fe electrode, COD, TOC, TN, and oil-grease removal efficiencies were determined as 76.3%, 71.8%, 70%, and 74%, respectively. Moreover, the highest COD, TOC, TN, and oil-grease removal efficiencies were achieved with an Al electrode (82.2%, 82.3%, 82.7%, and 78.9%, respectively). The experimental data were fit to a variety of isotherms and kinetic models to determine the characteristics of the EC. The results indicated that the pseudo-second-order equation provided the best fit for COD removal. Under optimum conditions, the operating cost was calculated as $3.39 and $3.09 for Al and Fe electrodes, respectively. In this study, the fuzzy axiomatic design method was used for the first time to select the most appropriate treatment method for PSW. In addition, blood, a major problem for the poultry slaughterhouse industry, was mixed with PSW at a ratio of 1% (v/v) and treated with EC for the first time with high removal efficiency. By treating PSW, which has a high pollution load, with electrocoagulation, the pollution load of the water to be given to secondary treatment has been greatly reduced.

## Introduction

Due to the rapid growth of the poultry industry, poultry slaughterhouses are producing increasing amounts of organic solid by-products and wastes (Salminen and Rintala 2002). Effluents from poultry slaughterhouse plants contain high levels of organic matter. The main contributors to the organic load of these effluents are paunch, feces, fat, lard, undigested food, blood, suspended material, urine, loose meat, soluble proteins, excrement, and particles (Al-Mutairi et al. 2004; Chávez et al. 2005; Massé and Masse 2000, 2001; Melo et al. 2008). The potential for pollution by the PSW is high due to the large amounts of waste produced in line production, including blood, which is difficult to degrade. Blood, oils, and greases give a red color to the slaughterhouse wastewater and are a significant problem in this type of effluent (Melo et al. 2008). Discharging PSW with a high organic pollution load into the receiving environment without treatment causes major environmental problems. In fact, this wastewater causes problems such as reducing the light transmittance in aquatic environments due to the blood and fat in its structure, negatively affecting the amount of dissolved oxygen and being a major food source for microorganisms. PSW treatment used processes such as coagulation (Dassey and Theegala 2012), electrocoagulation (Potrich et al. 2020), oxidation (Vidal et al. 2019), reverse osmosis (Reilly et al. 2019), anaerobic digestion (Loganath and Senophiyah-Mary 2020), dissolved air floatation, and membrane bioreactors (Goswami and Pugazhenthi 2020). Blood is the most problematic component in PSW due to its ability to inhibit floc formation during the activated sludge process. PSW is rich in oils, greases, sanitizers, and blood; however, to decrease the organic load of the PSW and to treat wastewater with biological systems, these substances must be removed by physicochemical treatment (Pozo et al. 2003, 2006; Pozo and Diez 2003). Oils, greases, sanitizers, and blood are long-chain organic compounds that require relatively long retention times in biological treatment processes to achieve complete oxidation (Sena et al. 2008). Therefore, to improve the efficiency of aerobic treatment processes, blood should not be combined with slaughterhouse wastewater. Alternatively, blood should be treated in the rendering process that converts waste animal tissue into stable, value-added materials. In rendering plants, the material is heated under pressure to kill microorganisms and to remove moisture (Sironi et al. 2007); however, this process requires large amounts of energy. In typical PSW, the blood volume is approximately 1% of the total volume of wastewater. DAF units are often used for the pretreatment of wastewater from poultry slaughterhouses (De Nardi et al. 2008); however, DAF systems cannot remove blood from wastewater. Alternatively, electrocoagulation is an effective method for the treatment of bloody wastewater from poultry slaughterhouses. Prior to aerobic biological treatment, electrocoagulation can be conducted to pretreat bloody wastewater, preventing the use of chemical-DAF systems.

Aerobic and anaerobic methods have been traditionally used for the treatment of PSW. Aerobic treatment processes are limited by high energy requirements, low oxygen transfer capacities, and the production of sludge (Chen and Shyu 1998; Pozo et al. 2003; Rinquest et al. 2019; Hilares et al. 2021a, b). Electrocoagulation (EC) has attracted a great deal of attention for industrial wastewater treatment due to its versatility and environmental compatibility. Moreover, EC can be used to treat a variety of liquids, gases, and solids. Coagulation, DAF, and anaerobic treatment techniques have limited application due to the high organic content of PSW. In particular, when PSW contains blood, coagulation and DAF systems cannot provide effective treatment. Therefore, innovative treatment methods such as EC have gained importance in blood containing PSW. EC is not selective in organic pollutants and produces small amounts of sewage sludge. Even with a short reaction time, it is effective in removing pollutants that are difficult to treat, such as blood (Kobya et al. 2003; Şengil et al. 2009; Şengil and Özacar 2006, 2009).

PSW treatment methods have advantages and disadvantages among themselves in terms of certain criteria. The axiomatic design method plays a decisive role in scientifically choosing the most suitable treatment method among these. Thus, random search processes are minimized in the selection of the treatment method, and the repeated attempts for process selection are also reduced (Suh 1990, 2001).

In this study, the fuzzy axiomatic design method was used to select the most appropriate treatment process for PSW treatment. Coagulation, DAF, electrocoagulation, and anaerobic treatment methods, which are widely preferred in PSW treatment, include initial investment cost, operating cost per pollution load removed, facility installed area, treatment time per pollutant load removed, pool volume, controllability of system conditions, volume of sludge formed. They were compared according to periodic maintenance frequency and oil-grease removal criteria. The optimum conditions for the treatability of PSW are the EC process, which is the most suitable method selected using fuzzy axiomatic design. Treatment of PSW containing 1% blood by EC process with Al electrode and Fe electrode has been examined for COD, TOC, and TN parameters. Conductivity, pH, current density, and reaction time parameters have been optimized for wastewater treatment in the EC process. In addition, the energy consumption of the processes was calculated for pH, current density, and reaction time. Both processes were compared for COD, TOC, and TN removal efficiencies and energy consumption under optimum conditions.

## Material and methods

### Characteristics of the PSW

The wastewater used in this study was obtained from the Şen Piliç poultry slaughterhouse of Sakarya (Turkey), which produces approximately 2500 m^3^ of wastewater per day. The raw PSW consisted primarily of organic compounds including carbohydrates, starches, proteins, suspended particles, clotted blood, and other ingredients. Poultry slaughterhouse wastewater was supplied from the stabilization pool. The daily amount of wastewater and the daily amount of blood released were determined, and 1% blood by volume was added to the PSW at the slaughterhouse. Then, the wastewater obtained from the facility was brought to the laboratory in a glass container with a volume of 30 L and stored at + 4 °C. The composition of the wastewater is shown in Table 1.

**Table 1:** Characteristics of PSW

Characteristics	Value
Chemical oxygen demand (COD) (mg L^−1^)	9300
Total organic carbon (TOC) (mg L^−1^)	2634
Oil and grease (mg L^−1^)	2300
Total phosphorus (mg L^−1^)	9.1
Total nitrogen (mg L^−1^)	1240
pH (at 25 °C)	6.7

### Axiomatic design

Axiomatic design is one of the design methods developed by Suh in order to make the design scientific for systems, products, and processes (Suh 1990). The main objectives of the axiomatic design method are to create a scientific basis for the designs, to minimize the random search processes, to reduce the repeated trials, and to decide on the most suitable design among the alternatives (Suh 2001). There are two design axioms considered in the axiomatic design method.

There are two design axioms considered in the axiomatic design method. The first of these, the independence axiom, argues that the independence of functional requirements (FR), which is defined as the minimum number of independent functional requirements that qualify the design objectives, should be maintained continuously. The knowledge axiom argues that the design with minimum information content is the most ideal among the designs that provide the independence axiom (Suh 1998).

The probability of satisfying a given functional requirement is defined by information content *I*. If the probability of satisfying a given FR is *p*, the information content *I* about the probability is expressed by Eq. 1:

1 $${I}_{i}={{\text{log}}}_{2}\frac{1}{{P}_{i}}$$

**Fig. 1:**
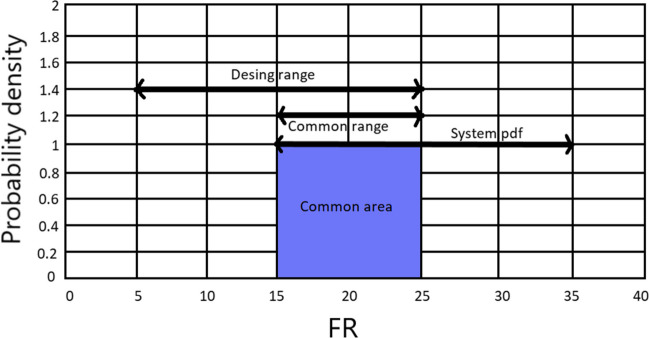
Probability density function of a FR

Since there are *N* Fi, the sum of the information content will equal the sum of all these probabilities. If one or more probabilities are 0, the information content is infinite. When the sum of all probabilities is 1, the information content is 0 (Pappalardo and Naddeo 2005). In order to calculate the probability of occurrence, the design range (*d*_r_) and system range (*s*_r_) must be determined. Figure 1 shows that when the system probability density function of a FR is uniform, the intersection of the designer’s “design range” and the system’s realized “system range” is the area where the acceptable solution is found. When the system probability density function is uniform, the probability of FR occurring is calculated by Eq. 2 (Kulak and Kahraman 2005):

2 $${P}_{i}=\frac{\mathrm{common\;range}}{\mathrm{system\;range}}$$

According to Eq. 2, the information content can be calculated from Eq. 3:

3 $${I}_{i}={{\text{log}}}_{2}\left(\frac{\mathrm{system\;range}}{\mathrm{common\;range}}\right)$$

**Fig. 2:**
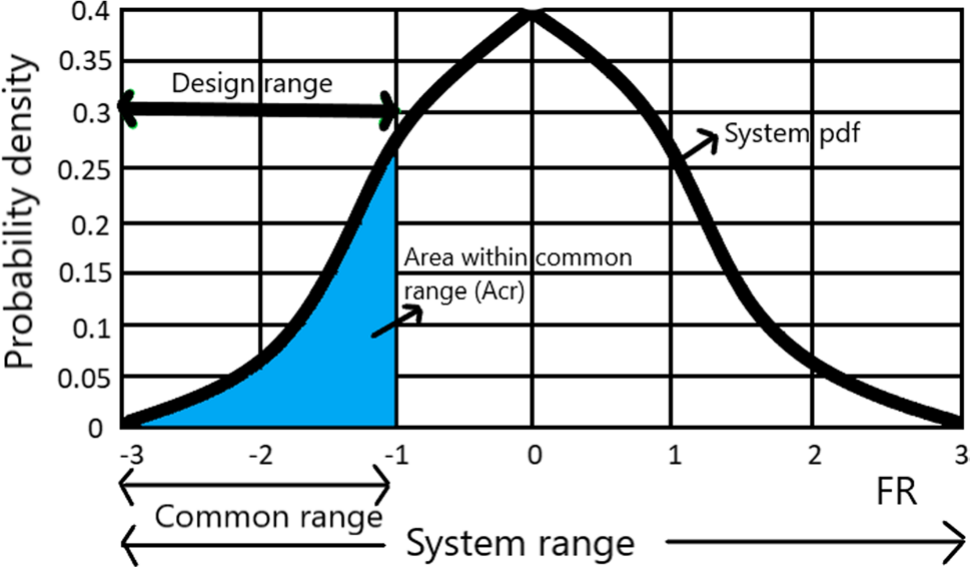
Probability density function of a FR expressed as a continuous random variable

In Eq. 4, the probability of realization of the interval of the whole system is calculated by integrating the probability density function of the system. Here, *d*^*ru*^ is the upper design range and *d*^*ri*^ is the lowest limit of the design range (Suh 2001). Figure 2 below shows the probability density function corresponding to a FR for which the system range is determined. Here, the shaded region common area (*A*_*cr*_) between the system range and the design range is the region where only the functional needs are met. Accordingly, the probability of the degree of realization of the determined goal of the design is found by dividing the area under the system gap by the area under the common gap (Özel and Özyörük 2007; Suh 1998).

For the regions seen in Fig. 2, Eq. 4 is obtained to express *A*_*sr*_ the area under the system interval and *A*_*cr*_ the shaded area under the common interval.

4 $${I}_{i}={{\text{log}}}_{2}\left(\frac{{A}_{sr}}{{A}_{cr}}\right)$$

Generally, since *A*_*sr*_ = 1, the information content is expressed by Eq. 5 since there are *n* FRs to be provided.

5 $${I}_{i}={{\text{log}}}_{2}\left(\frac{1}{{A}_{cr}}\right)$$

**Fig. 3:**
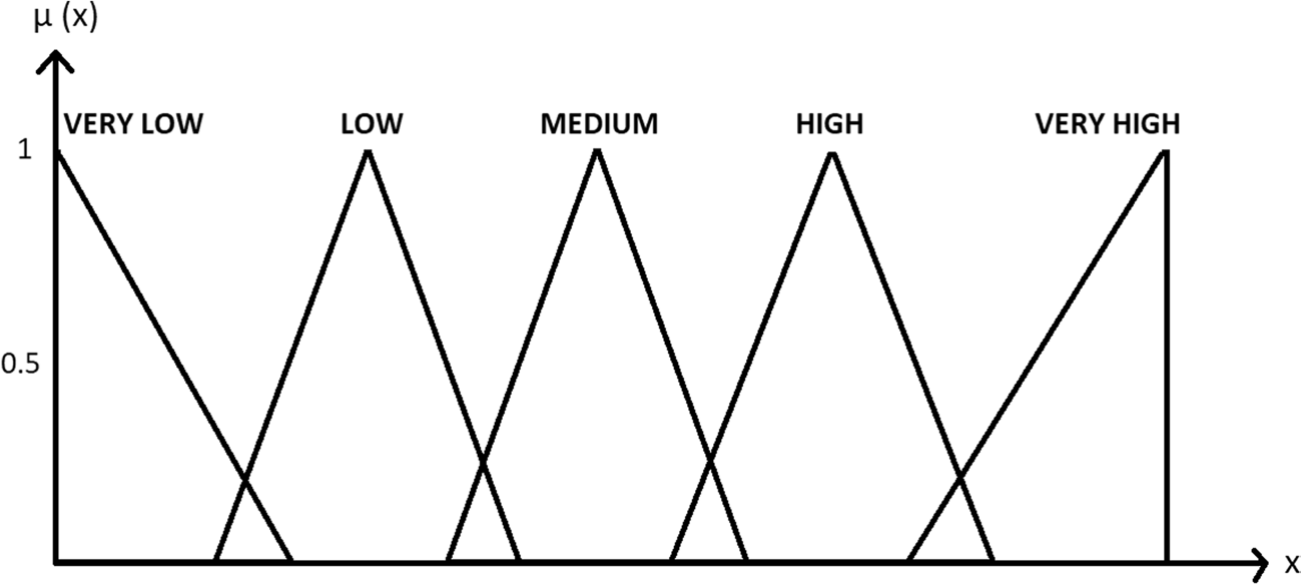
Fuzzy membership functions for linguistic expressions

When using multi-criteria decision-making methods, the data are known with certainty. Fuzzy axiomatic design is one of the methods used in cases where the data is not precise. Although real numbers are used when expressing data with certain values, linguistic variables are used in cases where there are no exact values. Using linguistic variables, data is converted into numerical form under certain rules. The most important tool used at this stage is fuzzy set theory. The representation of the membership functions of linguistic variables in order to digitize non-numeric values is as in Fig. 3 (Chen et al. 2018; Li 2012).

**Fig. 4:**
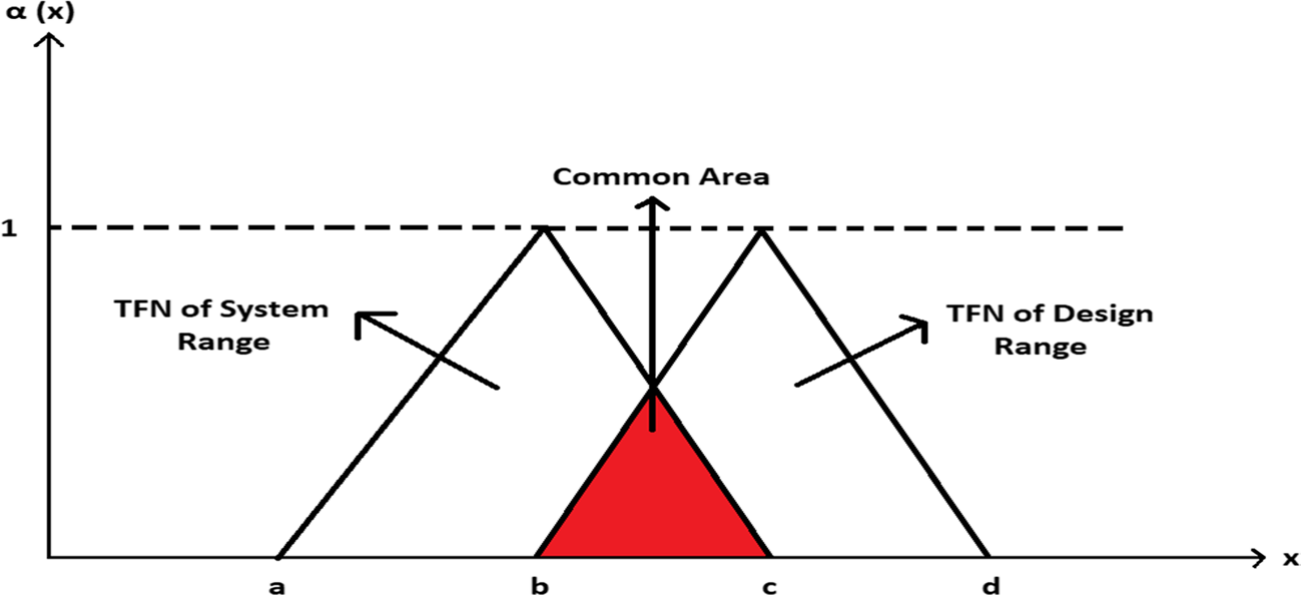
The common area of system and design ranges

In the case where AT is used and the data is fuzzy, trapezoidal or triangular fuzzy membership functions can be used when the range values of the design are given linguistically. Therefore, the common area will occur in the region where the trapezoidal or triangular fuzzy numbers (TFN) intersect. As seen in Fig. 4, the common area is formed at the intersection between the fuzzy triangular area of the design range and the fuzzy triangular area of the system gap (Gülümser et al. 2021).

Accordingly, the information content

6 $${I}_{i}={{\text{log}}}_{2}\left(\frac{\mathrm{triangular\;fuzzy\;area\;of\;system\;design}}{\mathrm{common\;area}}\right)$$

### EC reactor and experimental procedure

**Fig. 5:**
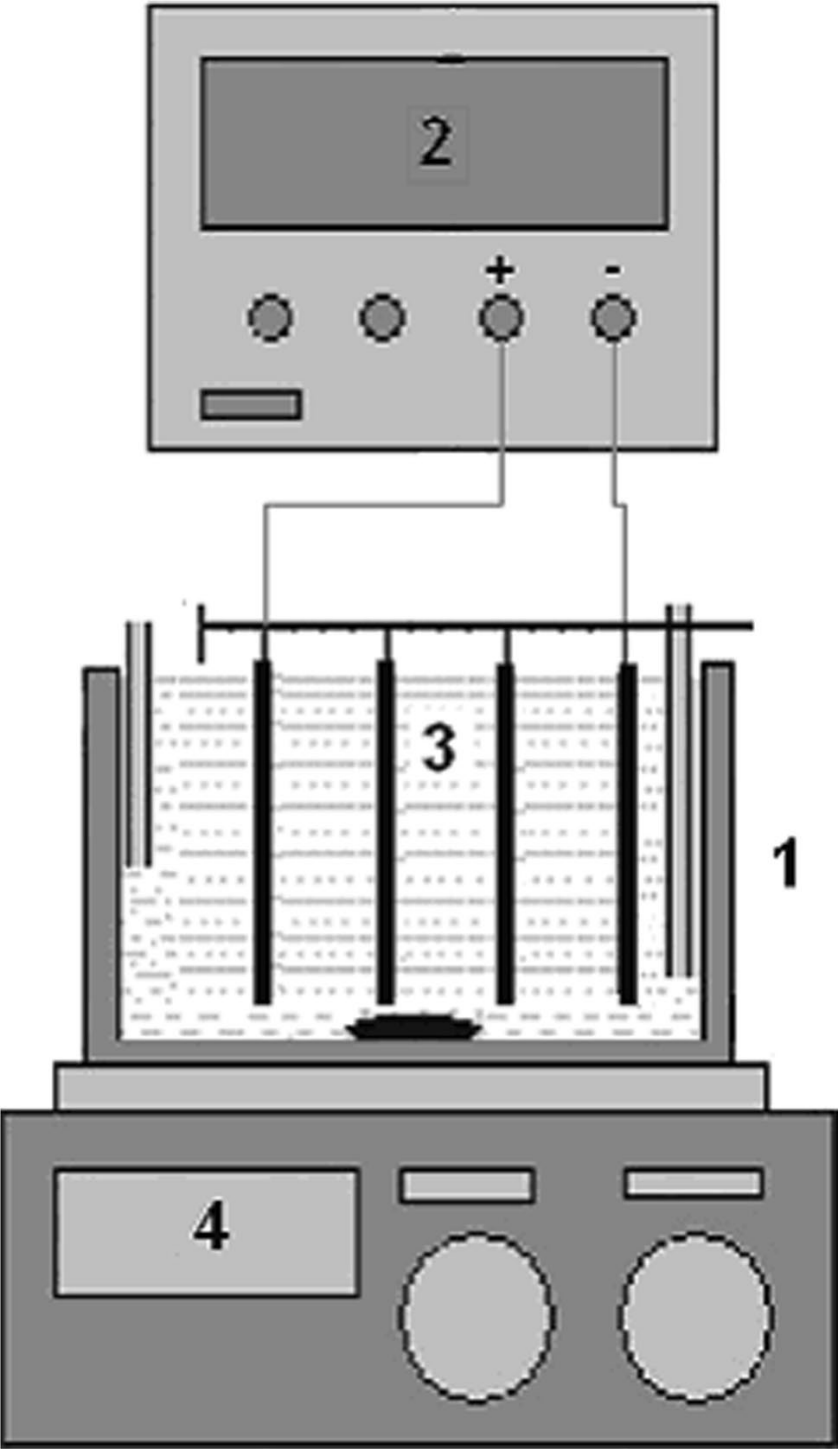
Schematic diagram of experimental setup (1: electrocoagulation cell, 2: DC power supply, 3: bipolar electrodes, 4: magnetic stirrer)

The experimental setup is shown in Fig. 5. The EC process consists of a 500 mL glass reactor, DC power supply (Yıldırım Electronic), magnetic stirrer (MTops), and iron or aluminum electrodes. Four electrodes with dimensions of 6 × 12 × 1.5 cm were used in the reactor, and these electrodes were connected bipolar fashion. The total effective electrode area was 288 cm^2^, and the spacing between the electrodes was 7.5 mm. A DC source was used to supply the system with 20 V and 10 A of power. A magnetic stirrer was employed to maintain thorough mixing of the PSW during the electrocoagulation process. Before each run, the electrodes were washed with pure water to remove surface grease, and impurities on the aluminum or iron electrode surfaces were removed by dipping the electrodes of a freshly prepared solution of 0.5 M HCl (Merck) (35%) for 3 min. The treatment of raw PSW wastewater was studied in a volume of 200 mL. At the end of the run, the solution was centrifuged (Nüve) and analyzed for TOC, TN, and COD. All experiments were performed in triplicate. And then, the analyzed samples were stored in disposable polypropylene (PP) plastic sample storage containers and stored at + 4 °C.

pH was measured with pH meter (Hanna). The PSW’s COD, TOC, and TN were measured according to standard methods (APHA, 2017). The parameters measured in the experiments were analyzed according to pH SM: 4500 H^+^ B, COD SM: 5220 C, TOC SM: 5310 B, TN SM: 4500-N E, and oil grease SM: 5520 B. The TOC and TN content of the PSW was measured with a HACH Model TOC/TN instrument (Hach Company). The pH was adjusted to the desired value with solutions of 0.5 M NaOH or 0.5 M H_2_SO_4_ (Merck).

## Results and discussion

### Axiomatic design

In the study, it is desired to choose between 4 different methods such as coagulation, electrocoagulation, DAF, and anaerobic treatment used to treat the slaughterhouse wastewater, under certain criteria. For this, the treatment method selection is discussed with the fuzzy axiomatic design method. In the study, 9 important criteria have been determined by considering the criteria affecting the selection of the treatment method; the articles examined and the opinions of the experts. These are initial investment cost (*C*_1_), operating cost per pollution load removed (*C*_2_), facility installed area (*C*_3_), treatment time per pollutant load removed (*C*_4_), pool volume (*C*_5_), controllability of system conditions (*C*_6_), volume of sludge formed (*C*_7_), periodic maintenance frequency (*C*_8_), and oil-grease removal (*C*_9_).

Electrocoagulation method has many advantages in terms of short retention time, less sludge formation, simple equipment requirement, and no chemical addition. However, it has some disadvantages such as electricity requirement and passivation as a result of contamination of the electrode plates (Asfaha et al. 2021). Although the coagulation process is a method that provides high treatment efficiency with the use of inorganic compounds, its use is limited due to the toxic nature of the sludge formed and the high chemical dosage required in large-scale applications (Hilares et al. 2021a, b). Aerobic treatment and anaerobic treatment methods, which are biological treatment processes, also have high removal efficiencies. Anaerobic treatment is more preferred than aerobic treatment. Because there is no need for ventilation in anaerobic treatment, energy consumption is at a minimum level and the volume of the treatment pool is also very small (Lima et al. 2020).

In order to apply the information axiom, the axiom of independence from the axiomatic design requirements must first be satisfied. In this application, it is assumed that the alternatives are independent of each other. In the fuzzy axiomatic design method, first of all, the design ranges of the criteria should be determined by experts. For this purpose, expert opinions were taken, and design intervals have been determined. After the design ranges of the criteria have been determined by the experts, the system ranges for each alternative’s criteria have been obtained and presented in Table 2. By determining the design and system ranges, the triangular fuzzy area and common area of the system design have been calculated with the GeoGebra program using the fuzzy member functions given in Fig. 3. System range area and common range area are given in Table 3.

**Table 2:** System and design ranges of criteria for alternative treatment methods in terms of TFNs

Criteria	Design range	System range			
		Coagulation	Electrocoagulation	Dissolved air flotation	Anaerobic treatment
*C*_1_	(0,0,14)	Low (2,4,6)	High (8,10,12)	Very low (0,0,4)	Medium (5,7,9)
*C*_2_	(0,0,14)	Medium (5,7,9)	Very low (0,0,4)	Medium (5,7,9)	Low (2,4,6)
*C*_3_	(0,0,14)	Medium (5,7,9)	Very low (0,0,4)	Medium (5,7,9)	Very high (10,14,14)
*C*_4_	(0,0,14)	Medium (5,7,9)	Very low (0,0,4)	Medium (5,7,9)	Very high (10,14,14)
*C*_5_	(0,0,14)	Medium (5,7,9)	Very low (0,0,4)	Medium (5,7,9)	Very high (10,14,14)
*C*_6_	(14,14,0)	Medium (5,7,9)	Very high (10,14,14)	Medium (5,7,9)	Very low (0,0,4)
*C*_7_	(0,0,14)	Medium (5,7,9)	Medium (5,7,9)	Medium (5,7,9)	Very low (0,0,4)
*C*_8_	(0,0,14)	Very low (0,0,4)	High (8,10,12)	Very low (0,0,4)	Medium (5,7,9)
*C*_9_	(14,14,0)	Very low (0,0,3)	High (8,10,12)	High (8,10,12)	Very high (10,14,14)

**Table 3:** System range areas and common range areas of alternative treatment methods (unit^2^)

Criteria	Coagulation		Electrocoagulation		Dissolved air flotation		Anaerobic treatment	
	System range area	Common range area	System range area	Common range area	System range area	Common range area	System range area	Common range area
*C*_1_	2	1.83	2	0.96	2	2	2	1.49
*C*_2_	2	1.49	2	2	2	1.49	2	1.83
*C*_3_	2	1.49	2	2	2	1.49	2	0.44
*C*_4_	2	1.49	2	2	2	1.49	2	0.44
*C*_5_	2	1.49	2	2	2	1.49	2	0.44
*C*_6_	2	1.49	2	2	2	1.49	2	0.44
*C*_7_	2	1.49	2	1.49	2	1.49	2	2
*C*_8_	2	2	2	0.96	2	2	2	1.49
*C*_9_	2	0.44	2	1.83	2	1.83	2	2

**Fig. 6:**
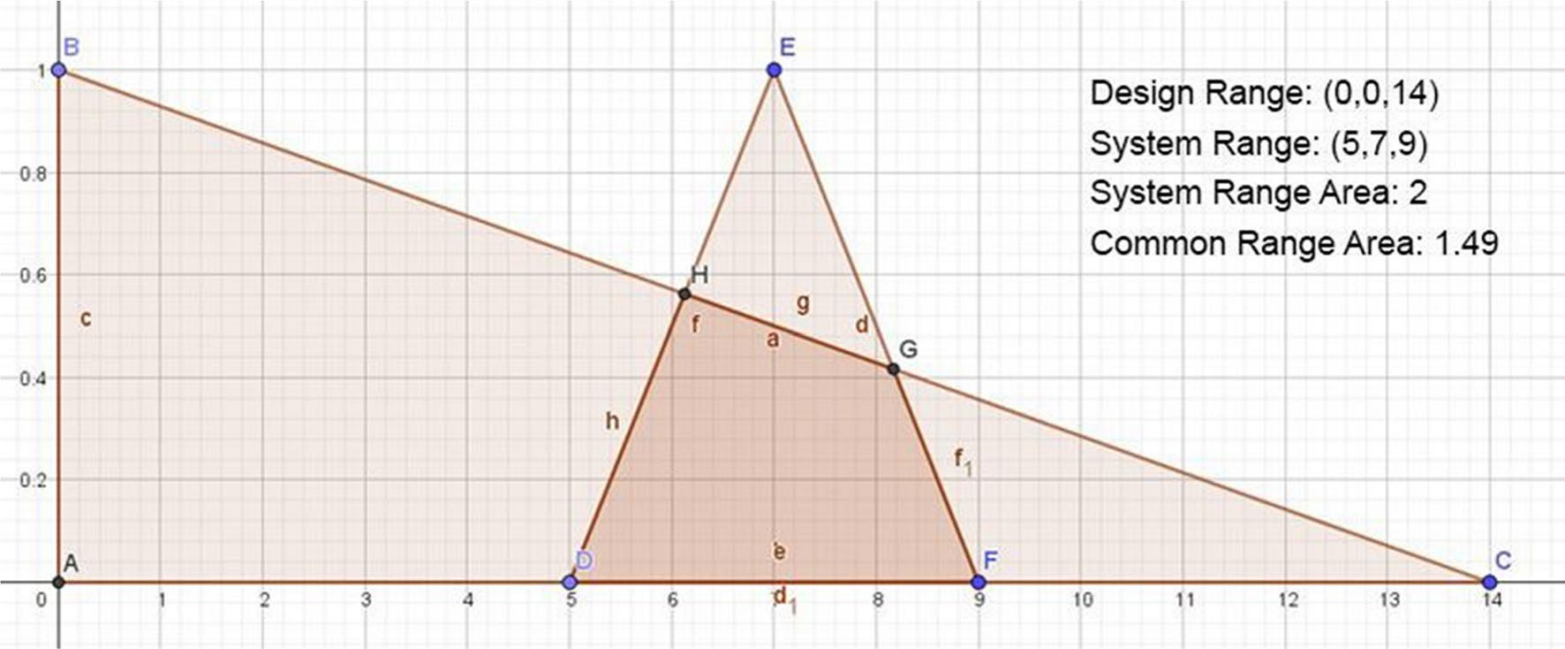
Information content in terms of the initial investment cost criterion for the anaerobic treatment method

Information content calculations can be made for each treatment method by using Eq. 7. For example, the information content in terms of the initial investment cost criterion for the anaerobic treatment method is calculated below and modeled in Fig. 6.

7 $${I}_{i}={{\text{log}}}_{2}\left(\frac{2}{1.49}\right)=0.425$$

The information content for all criteria of each alternative is calculated and shown in Table 4. In axiomatic design, the alternative that produces the smallest information content is considered the alternative that should be preferred. As can be seen from Table 4, the information content from the smallest to the largest is EC, DAF, coagulation, and anaerobic treatment, respectively. Considering these data, this method was chosen for the treatment of PSW, since the treatment process with the least information content is EC.

**Table 4:** Information contents for alternative treatment methods

Information contents	Coagulation	Electrocoagulation	Dissolved air flotation	Anaerobic treatment
*I*_C1_	0.128	1.059	0	0.425
*I*_C2_	0.425	0	0.425	0.128
*I*_C3_	0.425	0	0.425	2.184
*I*_C4_	0.425	0	0.425	2.184
*I*_C5_	0.425	0	0.425	2.184
*I*_C6_	0.425	0	0.425	2.184
*I*_C7_	0.425	0.425	0.425	0
*I*_C8_	0	1.059	0	0.425
*I*_C9_	2.184	0.128	0.128	0
∑	**4.862**	**2.671**	**2.678**	**9.714**

### Experiment study of EC for PWS

#### Effect of the supporting electrolyte

Conductivity is of particular importance in wastewater with low ionic strength. Because ions (anion and cation) in solution migrate easily with high conductivity at lower applied potential (Eryuruk et al. 2018), salts are required in electrochemical treatment processes to support the electrolyte and increase the solution’s conductivity. The conductivity of the solution affects the current efficiency, cell voltage, and the consumption of energy in electrolytic cells (Ghernaout et al. 2019; Graça et al. 2019). In the present study, a NaCl solution was used as the supporting electrolyte to increase the solution’s conductivity and reduce the consumption of electricity.

**Fig. 7:**
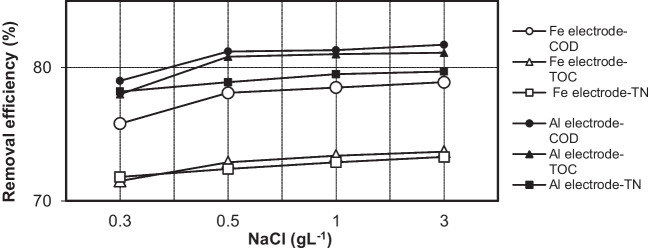
The effect of NaCl concentration on the COD, TOC, and TN removal by Fe and Al electrodes (*i* = 0.639 mA·cm^−2^; *t* = 5 min; *C*_*o*,COD_ = 9300 mg·L^−1^; *C*_*o*,TOC_ = 2634 mg·L^–1^; *C*_*o*,TN_ = 1240 mg·L.^–1^; *T* = 298 K; stirrer speed = 120 rpm; pH = 5; blood rate = 1%)

The effect of the NaCl concentration on the COD, TOC, and TN removal efficiency of iron and aluminum electrodes is shown in Fig. 7. The results indicated that the conductivity of the solution and the current density increased with an increase in the salt concentration. In general, an increase in the ionic strength at a constant cell voltage leads to an increase in the current density. Moreover, the cell voltage decreases with an increase in wastewater conductivity at a constant current density. Thus, as the ionic strength of the solution increases, the voltage required to attain a specific current density decreases, along with the overall consumption of electricity (Mollah et al. 2001, 2004).

As shown in Fig. 7, a significant improvement in the removal efficiency of COD, TOC, and TN was not observed for either electrode at a supporting electrolyte concentration greater than 0.5 mg·L^−1^. As the concentration of the supporting electrolyte increased from 0.5 to 3.0 mg·L^−1^, the COD, TOC, and TN removal rate of the Fe electrode increased from 78.1 to 78.9%, 72.9 to 73.7%, and 72.4 to 73.3%, respectively. The removal efficiency of COD, TOC, and TN for the Al electrode also increased slightly from 81.2 to 81.7%, 80.8 to 81.1%, and 78.9 to 79.7%, respectively. These results indicated that high removal efficiencies at low cell voltages could be obtained with a NaCl concentration of approximately 0.5 g·L^−1^. Thus, a NaCl concentration of 0.5 g·L^−1^ was used in future experiments.

#### Effect of initial pH

The pH of the solution is an important parameter affecting the performance of EC. The kinetics of the conversion of Fe^2+^ to Fe^3+^ are strongly affected by the pH, and the surface charge of coagulating particles is also pH-dependent (Song et al. 2007).

**Fig. 8:**
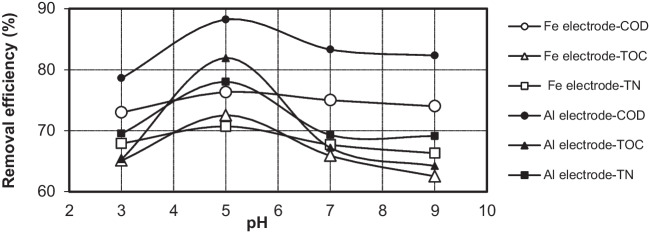
The effect of initial pH on the COD, TOC, and TN removal by Fe and Al electrodes (*i* = 0.639 mA·cm^−2^; *C*_*o*,COD_ = 9300 mg·L^−1^; *C*_*o*,TOC_ = 2634 mg·L^−1^; *C*_*o*,TN_ = 1240 mg·L^−1^; *t* = 5 min; NaCl = 0.5 g·L^−^.^1^; *T* = 298 K; stirrer speed = 120 rpm; blood rate 1%)

The effect of the initial pH on the removal efficiency of COD, TOC, and TN for Fe and Al electrodes is presented in Fig. 8, respectively. As shown in the figure, similar trends in the removal of COD, TOC, and TN were observed. As seen in Fig. 8, while there is an increase in COD, TOC, and TN removal efficiencies from pH 3 to pH 5, there is a decrease in removal efficiencies at values greater than pH 5. While COD, TOC, and TN removal efficiencies for the Al electrode at pH 3 were measured as 78.6%, 65.3%, and 69.5%, respectively, the removal efficiencies were determined as 73%, 65.1%, and 67.9%, respectively, for the Fe electrode. In addition, the COD, TOC, and TN removal efficiencies at pH 9 were determined as 82.3%, 64.2%, and 69.1% for the Al electrode and 74%, 62.5%, and 66.3% for the Fe electrode, respectively. After 5 min of electrolysis, the best results were obtained at a pH of 5 and a current of 0.639 mA·cm^−2^. The corresponding removal efficiency of COD, TOC, and TN with the Fe electrode was 79.7%, 72.5%, and 70.7%, respectively. Alternatively, with the Al electrode, the removal efficiency of COD, TOC, and TN was 88.2%, 81.9%, and 78%, respectively.

The various processes that occur during EC tend to buffer the liquid medium. It was reported by Bayramoğlu et al. (2006) that high COD removal was obtained for Fe and Al electrode in acidic environment. At pH 2 of PSW wastewater, maximum COD removal was found to be 93% with aluminum electrode and 85% with iron electrode, whereas COD removal was 70% for aluminum and 60% for iron electrode when original PSW was treated at its initial pH (6.7) of wastewater has been reported (Bayramoglu et al. 2006; Kobya et al. 2006).

**Fig. 9:**
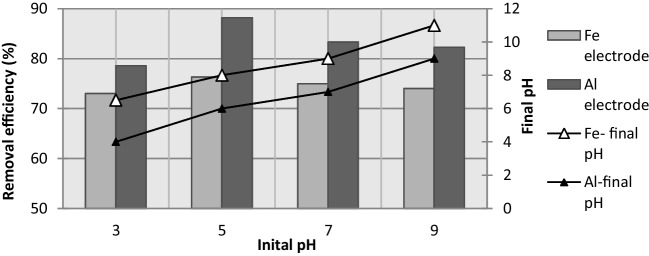
The effect of pH on the COD removal efficiency by Fe and Al electrodes (*i* = 0.639 mA·cm^−2^; *C*_*o*,COD_ = 9300 mg·L^−1^; *t* = 5 min; NaCl = 0.5 g·L^−^.^1^; *T* = 298 K; stirrer speed = 120 rpm; blood rate 1%)

The pH of the solution increases during the electrocoagulation process; however, the final pH of the solution after electrocoagulation is very important. As shown in Fig. 9, when the iron electrode was employed at an initial pH of 5, the final pH of the solution was equal to 8. Alternatively, after electrocoagulation with the Al electrode at an initial pH of 5, the final pH of the solution was equal to 6. With the Fe electrode, the maximum COD removal was attained at a final pH of 8, and the greatest amount of Fe(H_2_O)_4_(OH)_2(s)_ and Fe_2_O_3_(H_2_O)_6(s)_ flocculation was observed. Alternatively, the maximum COD removal by the Al electrode was observed at a final pH of 6. Depending on the pH, aluminum can form a variety of different species in solution. At a pH between 5 and 6, the predominant products of aluminum hydrolysis are Al(OH)_2_^+^ and Al(OH)^2+^. Alternatively, at a pH between 5.2 and 8.8, Al(OH)_3_ is the most prevalent species. At high pH values, Al(OH)_3_ dissolves in water, forming hydroxo-complexes [Al(OH)_*n*_]^−(*n*−3)^.

#### Effect of the current density

**Fig. 10:**
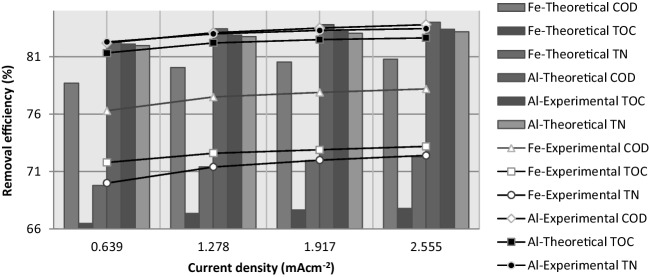
The effect of current density on the COD, TOC, and TN removal by Fe and Al electrodes (pH = 5; *C*_*o*,COD_ = 9300 mg·L^−1^; *C*_*o*,TOC_ = 2634 mg·L^–1^; *C*_*o*,TN_ = 1240 mg·L^–1^; *t* = 5 min; NaCl = 0.5 g·L^−^.^1^; *T* = 298 K; stirrer speed = 120 rpm; blood rate 1%)

The current density determines the coagulant production rate and alters the rate of bubble production, which affects the growth of flocs (Mollah et al. 2001). Figure 10 shows the effects of the current density on the removal efficiency of COD, TOC, and TN for iron and aluminum electrodes at an operating time of 5 min and a pH of 5. As the applied current density of the Fe electrode increased from 0.639 to 2.555 mA·cm^−2^, the removal efficiency of COD, TOC, and TN increased slightly from 76.3 to 78.2%, 71.8 to 73.2%, and 70 to 72.4%, respectively. The removal efficiencies of the Al electrode were greater than those of the Fe electrode. Moreover, as the current density increased from 0.639 to 2.555 mA·cm^−2^, the removal efficiency of COD, TOC, and TN increased from 82.2 to 83.8%, 82.3 to 83.4%, and 82.7 to 83.4%, respectively. The degree of anodic dissolution increases at high current densities and with it the amount of hydroxo-cationic complexes increases, resulting in an increase in impurity removal (Vepsäläinen and Sillanpää 2020). With the increase of the flow density, the removal efficiency of PSW wastewater increases. According to Bayar et al., approximately 85% COD removal efficiency was obtained at 0.5 mA·cm^−2^, pH 4 (Bayar et al. 2011).

The results of this study indicated that the amount of COD, TOC, and TN removed per unit weight of metal ion formed during the electrocoagulation process was linearly related to the residual concentration of the pollutant in solution. A general expression describing the global adsorption isotherm was developed:

8 $$\frac{\Delta C}{m}=k+n\cdot {C}_{{\text{res}}}$$

where *ΔC* is the concentration (mg·L^−1^) of the pollutant removed from the wastewater, *m* is the mass of dissolved Fe^2+^ or Al^3+^ formed during electrolysis (g·L^−1^), *C*_res_ is the residual concentration of the pollutant (mg·L^−1^) after electrocoagulation, *k* (mg·g^−1^) is a coefficient describing the removal capacity of the metal (in milligram of pollutant per gram of metal ion), and *n* (L·g^−1^) is another coefficient. Equation 8 is based on the mass balance of the pollutant (concentration removed = initial concentration − residual concentration). The value of *m* was calculated according to Faraday’s law, and the values of *k* and *n* were calculated from the slope and intercept of the linear plot of *ΔC*/*m* vs. *C*_res_ and are shown in Table 5.

**Table 5:** Isotherm model constants and correlation coefficients

Parameters	Fe			Al		
	*k*	*n*	*R*^2^	*k*	*n*	*R*^2^
COD	− 3,000,000	1745	0.997	− 10,000,000	6977	0.991
TOC	− 2,000,000	2390	0.991	− 4,000,000	8984	0.993
TN	− 434,622	1304	0.993	− 2,000,000	9835	0.993

The relationship between Eq. 9 and Faraday’s equation was derived from the following equations:

9 $${C}_{{\text{res}}}=\frac{{C}_{o}-k\left(\frac{I\cdot t}{z}\cdot \frac{M}{F}\right)}{1+n\left(\frac{I\cdot t}{z}\cdot \frac{M}{F}\right)}$$

10 $${C}_{{\text{res}}}=\frac{({C}_{o}\cdot F\cdot z)-(k\cdot i\cdot A\cdot t\cdot M\cdot 1{0}^{-3})}{(F\cdot z)+(n\cdot i\cdot A\cdot t\cdot M\cdot 1{0}^{-3})}$$

where *C*_*o*_ (mg·L^−1^) is the initial concentration in the wastewater, *I* is the current (A), *i* (mA·cm^−2^) is the current density, *A* (cm^2^) is the surface area of the electrode, *t* (s) is the time, *F* is Faraday’s constant (coulomb), *z* is the valence number, and *M* is the atomic weight (g·mol^−1^).

The theoretical values of the COD, TOC, and TN content for various values of *i* were calculated according to Eq. 10. Figure 10 shows a comparison of the theoretical results and the experimental data. In general, the calculated values were in good agreement with the experimental data.

**Fig. 11:**
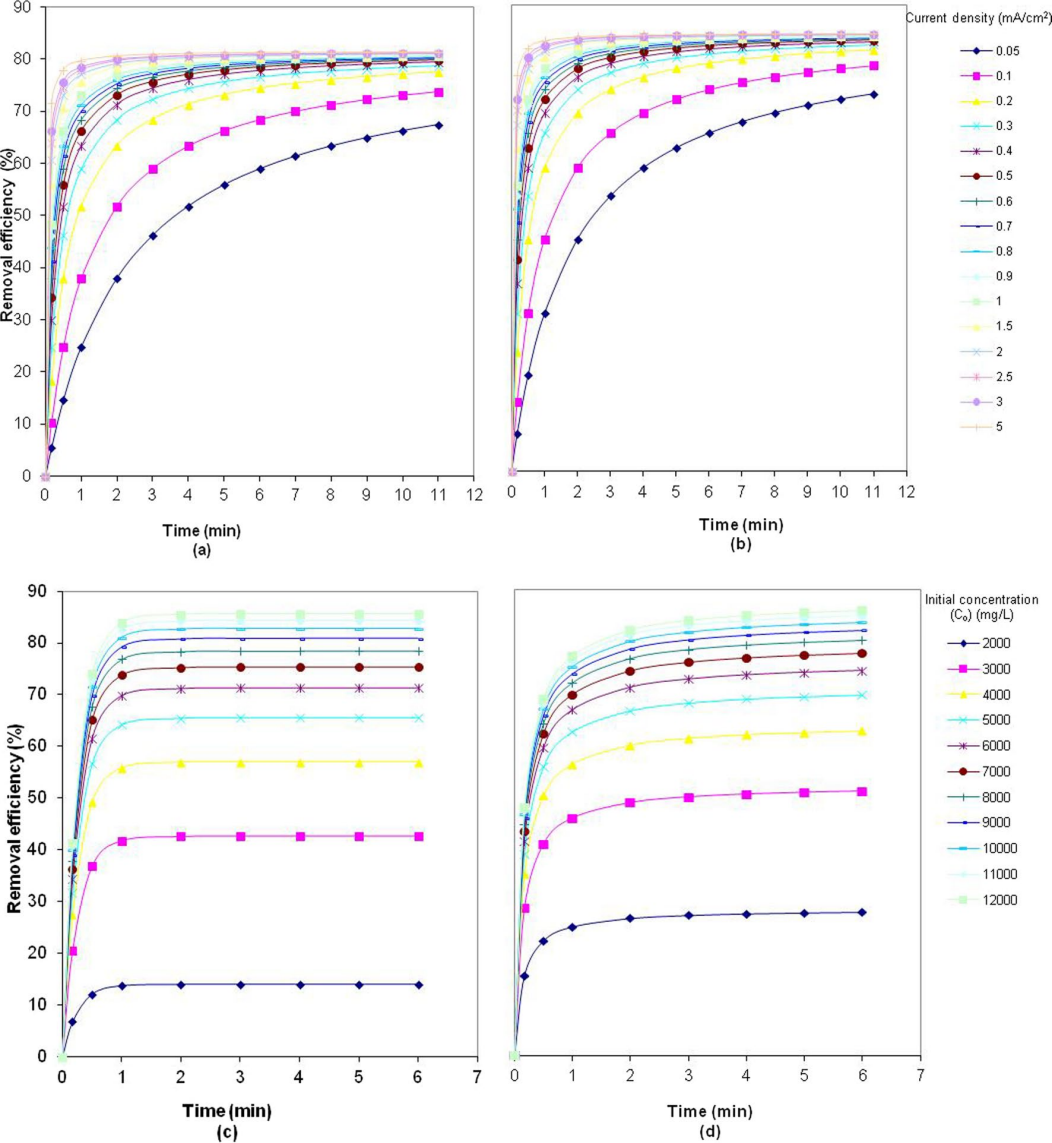
Numerically generated curves for COD by Fe and Al electrodes. **a**, **b** Numerically generated curves for COD removal opposition to current density by Fe and Al electrodes (pH = 5; *C*_*o*,COD_ = 9300 mg·L^−1^; NaCl = 0.5 g·L^−1^; *T* = 298 K; stirrer speed = 120 rpm; blood rate = 1%). **c**, **d** Numerically generated curves for COD removal opposition to initial concentration by Fe and Al electrodes (pH = 5; *i* = 0.639 mA·cm^−2^; NaCl = 0.5 g·L^−^.^1^; *T* = 298 K; stirrer speed = 120 rpm; blood rate = 1%)

The effect of the current density of the Fe and Al electrode at a constant COD (*C*_*o*_) was evaluated, as well as the effect of *C*_*o*_ at constant current density (a current density of 0.639 mA·cm^−2^ was chosen arbitrarily), and the results are shown in Fig. 11.

As shown in Fig. 11a, b, similar relationships between the electrolysis time and the removal efficiency of the Al and Fe electrodes were observed. With the Fe electrode, the removal efficiency increased from 55.9 to 81% as the current density increased from 0.05 to 5 mA·cm^−2^. Similarly, with the Al electrode, the removal efficiency increased from 62 to 84% as the current density increased from 0.05 to 5 mA·cm^−2^. At current densities greater than 0.64 mA·cm^−2^, the COD removal efficiency of aluminum and iron electrodes reached a maximum value of 82% and 79% at an electrolysis time of 5 min, respectively.

Figure 11c, d shows the effects of the initial COD concentration on the COD removal efficiencies of iron and aluminum electrodes at various initial COD concentrations. The results indicated that the COD removal rate of both electrodes increased with an increase in the initial concentration of COD because organic compounds present in the PSW were removed upon reaction with iron and aluminum ions, forming insoluble compounds. Furthermore, as the initial COD concentration increased, the time required to achieve complete electrocoagulation decreased. The reaction time required to remove more than 80% of the COD in wastewater with the initial COD of 9000 mg·L^−1^ is 5 min for both electrodes.

#### Effect of electrolysis time

**Fig. 12:**
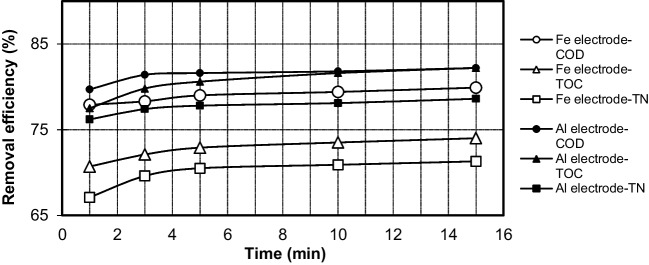
The effect of electrolysis time on the COD, TOC, and TN removal by Fe and Al electrodes (*i* = 0.639 mA·cm^−2^; *C*_*o*,COD_ = 9300 mg·L^−1^; *C*_*o*,TOC_ = 2634 mg·L^−1^; *C*_*o*,TN_ = 1240 mg·L^−1^; NaCl = 0.5 g·L^−^.^1^; *T* = 298 K; stirrer speed = 120 rpm; pH = 5; blood rate = 1%)

The electrolysis time (*t*) determines the rate at which ions are produced from the electrodes. Figure 12 shows the relationship between the removal efficiency of COD, TOC, and TN and the electrolysis time for both electrodes. The removal efficiency is dependent on the concentration of hydroxyl and metal ions produced from the electrodes. As shown in the figure, as the electrolysis time increased, significant changes in the removal efficiencies of both electrodes were not observed. At the end of the 1-min reaction time, the COD removal efficiency was achieved 79.7% for the Al electrode and 77.9% for the Fe electrode. In addition, TOC removal efficiencies were obtained as 77.5% and 70.7%, and TN removal efficiencies were 76.2% and 67.1% for Al and Fe electrodes, respectively. An approximately 2.5% increase in COD, TOC, and TN removal efficiencies was observed in both Al and Fe electrodes from 1-min reaction time to 5-min reaction time. Most of the pollutants were removed during the first 5 min of electrolysis, and further electrogenerated of coagulant flocs did not positively affect the removal efficiency. According to the results shown in Fig. 12, the optimal electrolysis time for the removal of COD, TOC, and TN from PSW was 5 min.

### Kinetic analysis

By using experimental data, reaction rate constants (*k*) and regression coefficients (*R*^2^) are determined by kinetic modeling (Kaur et al. 2020). A pseudo-second-order kinetic model for COD removal efficiencies depending on reaction time was calculated according to the equation below (Tanatti 2022).

11 $$\frac{t}{C}=\frac{1}{{k}_{2}{{C}_{e}}^{2}}+\frac{1}{{C}_{e}}\cdot t$$

where *C* is the residual pollutant concentration in the solution at time *t*, *C*_*e*_ is the concentration coefficient, *k*_2_ is the reaction rate coefficient, and *t* is the time.

The *k* and *R*^2^ values obtained from the pseudo-second-order kinetic model for Al and Fe electrodes are given in Table 6. As seen in Table 6, the *R*^2^ value was calculated as 0.9998 for the Al electrode and 0.9997 for the Fe electrode. The model is meaningful since the *R*^2^ values of the pseudo-second-order kinetic model are very close to 1 for both electrodes (Isgoren et al. 2017).

**Table 6:** The coefficients of the pseudo-second-order kinetic model

Parameter	*C*_*e*_ (mg L^−1^)	*k*_2_ (L mg^−1^ s^−1^)	*R*^2^
Fe electrode	2000	− 0.0068	0.9997
Al electrode	1666.7	− 0.0062	0.9998

### Energy consumption

One of the most important parameters that can affect the application of wastewater treatment methods is the operating cost. The amount of electrical energy consumed kWh·kg COD^−1^ during the removal of 1 kg of COD from a PSW by EC at a constant applied current was calculated as a function of time according to the following equation:

12 $${\text{EEC}}=\frac{\int U\cdot I\cdot dt}{({C}_{o,{\text{COD}}}-{C}_{t,{\text{COD}}})\cdot V\cdot 3.6}=\frac{I\int U\cdot dt}{({C}_{o,{\text{COD}}}-{C}_{t,{\text{COD}}})\cdot V\cdot 3.6}$$

where *U* is the applied voltage (V), *I* is the current (A), *t* is the electrolysis time (min), *C*_*o*,COD_ (mg·L^−1^) is the initial concentration of COD, *C*_*t*,COD_ (mg·L^−1^) is the COD concentration at time *t*, and *V* (L) is the volume of treated wastewater.

**Fig. 13:**
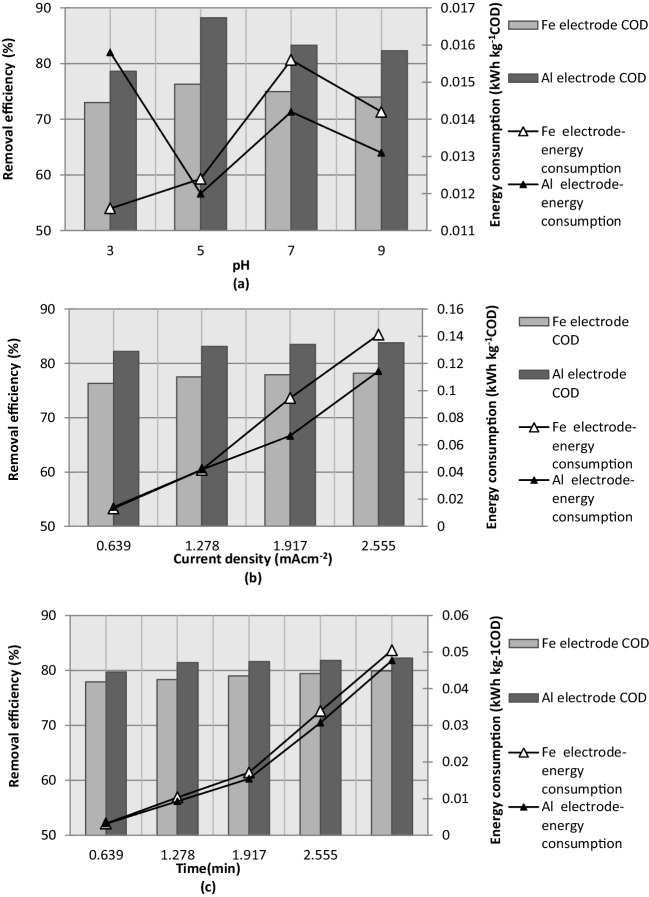
Effect of pH (**a**), current density (**b**), and electrolysis time (**c**) on the electric energy consumption and COD removal efficiency (*i* = 0.639 mA·cm^−2^; *C*_*o*,COD_ = 9300 mg·L^−1^; *t* = 5 min; NaCl = 0.5 g·L^−^.^1^; *T* = 298 K; stirrer speed = 120 rpm; blood rate = 1%)

The amount of energy consumed by the aluminum and iron electrodes is shown in Fig. 13a as a function of the pH of the solution. The lowest energy consumption curve for both electrodes was obtained at pH 5 because the COD removal rate is the highest at this pH. The reduction of 1.0 kg of COD at pH 5 consumed 0.012 kWh of energy, and the corresponding COD removal efficiencies of the Al and Fe electrode were 88% and 76%, respectively.

In Fig. 13b, the amount of energy consumed during the process was plotted as a function of the current density. The minimum energy consumption of the Fe and Al electrodes for COD removal was 0.0135 kWh·m^−3^ and 0.0141 kWh·m^−3^ and was observed at a current density of 0.639 mA·cm^−2^, respectively. The energy consumption of the iron and aluminum electrodes was nearly identical at a current density of 0.639 mA·cm^−2^; however, the COD removal efficiency of the Al electrode was slightly higher than that of the Fe electrode.

Figure 13c shows the effect of electrolysis time on the COD removal efficiency of a PSW containing 1% blood, as well as the amount of energy consumed during the process. As the electrolysis time increased from 1 to 15 min, the energy consumption of the Fe and Al electrode increased significantly from 0.003 to 0.0504 kWh·kg COD^−1^ and 0.003 to 0.0477 kWh·kg COD^−1^, respectively. Furthermore, the COD removal efficiency of the Fe and Al electrode increased from 78 to 79.9% and 80 to 82.2%, respectively. Thus, the best compromise between the COD removal rate and the amount of energy consumed during the process was observed at an electrolysis time of 5 min, providing a reasonable COD removal rate and relatively low energy consumption for both electrodes.

### Cost analysis

The operating cost has been calculated in the treatment of poultry slaughterhouse wastewater with EC using Al and Fe electrodes. The electrical energy, the amount of electrodes, the chemicals required for pH adjustment, and the amount of salt used for conductivity are important while determining the operating costs in the EC process. In the EC process, the operating cost required for the treatment of 1 m^3^ wastewater with Al and Fe electrodes was calculated in dollars. The electrical energy consumed in the EC process using Al electrode is 0.0141 kWh·m^−3^, and the electricity cost of the process is 0.00131 $·m^−3^. In the EC process using Fe electrode, the electrical energy consumed is 0.0135 kWh·m^−3^ and the electricity cost of the process is $0.00126 per 1 m^3^ of water. The amount of NaCl consumed in both processes is 0.5 g·L^−1^. The price of NaCl used for 1 m^3^ wastewater treatment is $2.26. In the EC process using Fe and Al electrodes, 0.25 mL·L^−1^ HCl is required for pH adjustment. The price of HCl required for 1 m^3^ of wastewater is $0.78. In the EC process, the electrode consumption is 0.06 g·L^−1^for the Fe electrode and 0.06 g·L^−1^ for the Al electrode. For the treatment of 1 m^3^ of wastewater, the required Fe electrode price is $0.054 and the required Al electrode price is $0.35. As a result, the total operating cost of the EC process using Fe electrode was determined as $3.09 per m^3^ wastewater. Moreover, in the EC process using Al electrode, the operating cost for the treatment of 1 m^3^ of wastewater was determined as $3.39. In the EC process using Fe and Al electrodes, the operating costs are close, but the cost is $0.30 less when Fe electrode is used. However, considering the COD removal efficiency in poultry slaughterhouse wastewater, using Al electrodes becomes a priority.

## Conclusions

In this study, 9 different criteria for PSW treatment processes with fuzzy axiomatic design method were compared among themselves. The information content from the smallest to the largest is EC, DAF, coagulation, and anaerobic treatment, respectively. According to the fuzzy axiomatic design method since the smallest information content is in the EC process, EC was a preferred treatment method. Removal of blood is very difficult in the poultry slaughterhouse industry. Blood from slaughterhouses has a high organic content and is a substance that is difficult to degrade. In addition, although blood can be removed traditionally with decanters in the slaughterhouse industry, it is a very costly practice. In this study, the removal of COD, TOC, and TN from wastewater by adding 1% of the volume of blood to the wastewater was examined using Al and Fe electrodes. Optimum conditions were determined by studying the supporting electrolyte concentration, pH, current density, and reaction time parameters of Al and Fe electrodes. The optimum conditions were found to be 0.5 g·L^−1^ NaCl concentration, pH 5, 0.639 mA·cm^−2^ current density, and 5 min time for both electrodes. Under optimum conditions, COD, TOC, TN, and oil-grease removal efficiencies were obtained as 82.2%, 82.3%, 82.7%, and 78.9% for the Al electrode and 76.3%, 71.8%, 70%, and 74% for the Fe electrode, respectively. The results of the present study revealed that EC is a suitable method for the removal of COD, TOC, TN, and oil/grease from bloody poultry slaughterhouse wastewater containing 1% blood by volume. Moreover, when Al electrode and Fe electrodes were compared in terms of COD, TOC, TN, and oil-grease removal efficiency, it was concluded that the Al electrode was more effective. Under optimum conditions, the operating cost was calculated as $3.39 and $3.09 for Al and Fe electrodes, respectively. With this study, the treatment of poultry wastewater containing blood is carried out on a laboratory scale, providing important results for practice. In addition, EC is a treatment process that has been studied for many industrial wastewaters and has some pilot applications. EC has many advantages over traditional treatment (for example, small treatment pools, short reaction time, low chemical requirement, and low energy requirement). Therefore, the EC process is an alternative treatment process that can replace traditional chemical treatment processes of poultry slaughterhouse wastewater. Moreover, it was concluded that EC could be a treatment process with potential for large-scale application. In summary, in this study, the most suitable treatment process was determined for the first time by applying the fuzzy axiomatic design method to the alternatives used in PSW treatment. Furthermore, blood, which is very difficult to remove, was added to wastewater and treated with EC for the first time.

## Data Availability

All relevant data are within the paper.
